# The Shiny Cowbird, *Molothrus bonariensis* (Gmelin, 1789) (Aves: Icteridae), at 2,800 m asl in Quito, Ecuador

**DOI:** 10.3897/BDJ.4.e8184

**Published:** 2016-05-03

**Authors:** Verónica Crespo-Pérez, C. Miguel Pinto, Juan Manuel Carrión, Rubén D. Jarrín-E, Cristian Poveda, Tjitte de Vries

**Affiliations:** ‡ Pontificia Universidad Católica del Ecuador, Escuela de Ciencias Biológicas, Av. 12 de Octubre y Roca, Quito, Ecuador; § Instituto de Ciencias Biológicas, Escuela Politécnica Nacional, Quito, Ecuador; | Smithsonian Institution, National Museum of Natural History, Division of Mammals, 1000 Constitution Ave NW, Washington DC, United States of America; ¶ Fundación Zoológica del Ecuador, Quito, Ecuador

**Keywords:** Brood parasite, Climate change, Habitat fragmentation, *Molothrus bonariensis*, Rufous-collared Sparrow, Shiny Cowbird, university campus, *Zonotrichia capensis*

## Abstract

**Background:**

The Shiny Cowbird, *Molothrus bonariensis* Gmelin, 1789, is a brood parasite of hundreds of small-bodied birds that is native to South American lowlands. Within the last 100 years this species has been expanding its range throughout the Caribbean, towards North America, but has rarely been seen above 2,000 m asl.

**New information:**

Here, we present records of Shiny Cowbirds in Quito, a city located 2,800 m above sea level that harbors a bird community typical of the Andean valleys. We found two juvenile individuals parasitizing two different pairs of Rufous-collared Sparrow (*Zonotrichia capensis* Müller, 1776). This report constitutes an altitudinal range expansion of reproductive populations of ca. 500m, which may have beenprompted by anthropogenic disturbance.

## Introduction

**Figure 1.**
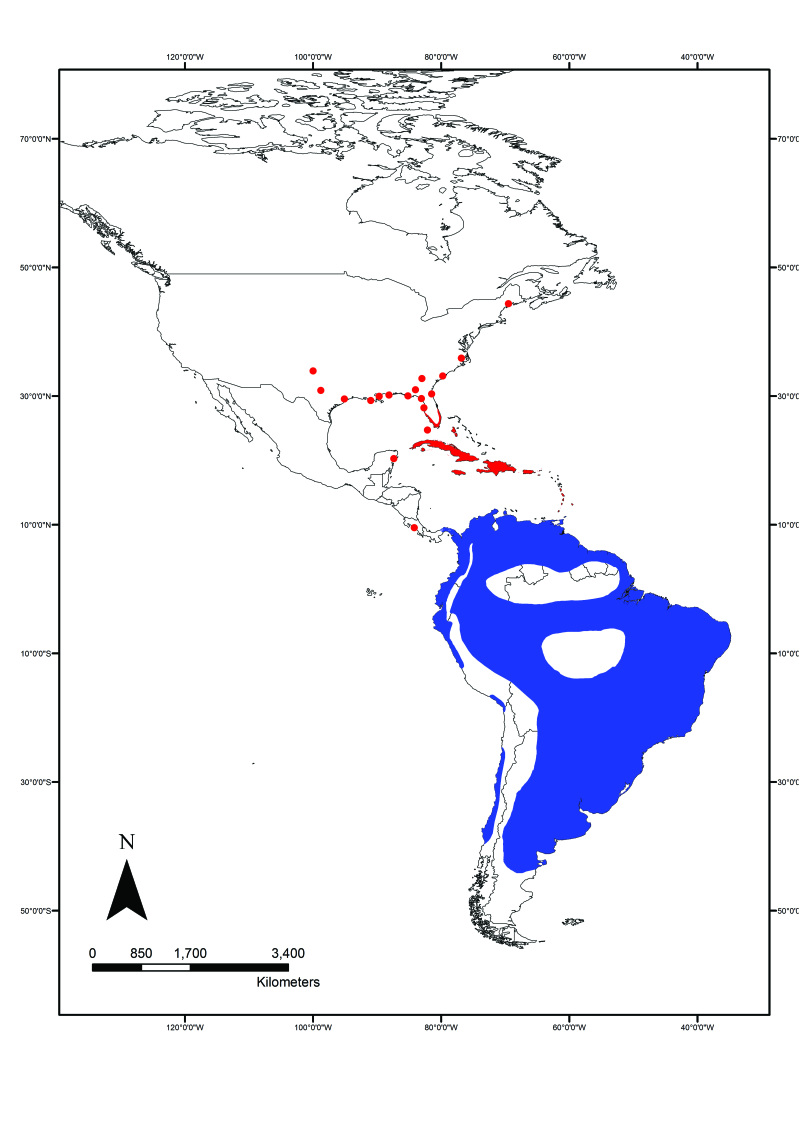
Distribution of the Shiny Cowbird in the Americas, including the Caribbean, modified from Post et al. (1993) and BirdLife International and NatureServe (2014). Areas in blue represent the native range, while areas in red represent the invaded range.

**Figure 2.**
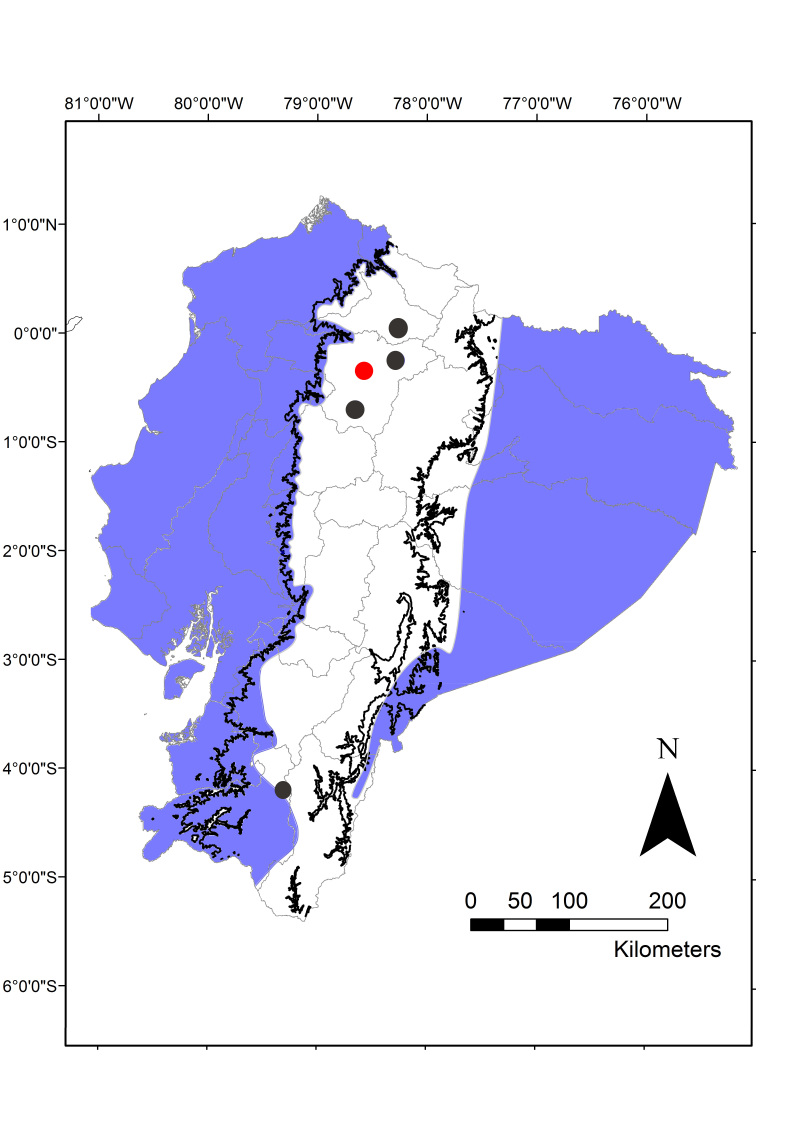
Distribution of the Shiny Cowbird in Ecuador, modified from Ridgely and Greenfield (2006). Areas in blue represent areas below 2000 m asl where the shiny cowbird has been previously reported (Ridgely and Greenfield 2006), black dots represent isolated, higher altitude records (up to 2700 m asl) (Ridgely and Greenfield 2006, Xeno-canto Foundation 2012), and the red dot marks the city of Quito, where we report the presence of the Shiny Cowbird.

The Shiny Cowbird, *Molothrus bonariensis* Gmelin, 1789, is a brood parasite of hundreds of small-bodied birds, and the Rufous-collared Sparrow, *Zonotrichia capensis* Muller, 1776 is one of its main hosts (Mason 1986, Lowther 2013). It is native to South American lowlands (Bird Life International and NatureServe 2012), but within the last 100 years has been expanding its distribution throughout the Caribbean, towards North America (Post et al. 1993) with some isolated records from Costa Rica and Mexico (Kluza 1998, Sandoval et al. 2010) (Fig. 1). The species occupies lowlands and it has rarely been seen above 2,000 m asl (Hilty and Brown 1986, Lowther 2011); however, there are notorious records in Bolivia and Peru above 3,000 m asl (Miller 1917, Fjeldsa and Krabbe 1990, Jaramillo and Burke 1999, Balderrama 2006, Schulenberg et al. 2007). Particularly in Ecuador, it has been mostly recorded between 900 and 1,000 m asl, but also regularly higher to ca. 1,400 to 1,600 m asl (Ridgely and Greenfield 2001, Ridgely and Greenfield 2006). There have been isolated records in the highlands (as high as 2,700 m asl) but there are no reports of these birds breeding at such altitudes. There also are records in the Pichincha province (up to 2,700 m asl) (Fjeldsa and Krabbe 1990, Ridgely and Greenfield 2001), and vocal recordings available from the southern city of Loja (2,100 m asl) and from lake San Pablo in the north of the country (2,700 m asl) in the sound library Xeno-Canto (www.xeno-canto.org) (Fig. 2). In the year 2000 the Shiny Cowbird was first spotted in the inter Andean valley of Cumbayá, at 2,300 m asl, 7 km to the east of Quito, Ecuador (Carrion 2001, Carrion pers. obs.). These birds are now regular inhabitants of that area and may be observed there year round, sometimes in groups of more than 50 individuals (Carrion pers. obs.).

Here, we present records of juvenile Shiny Cowbirds at 2,800m asl in Quito—Ecuador’s capital city located in the inter Andean valley, with a population of over 1.6 million people. The bird community of the city is typical of the inter Andean valleys (Carrion 2001), with many species of hummingbirds and other species like the Eared Dove, *Zenaida auriculata* Des Murs, 1847, the Golden Grossbeak, *Pheucticus chrysogaster* Lesson, 1832, the Great Thrush, *Turdus fuscater* Lafresnaye & d'Orbigny, 1837, the Vermilion Flycatcher, *Pyrocephalus rubinus* Boddaert, 1783, and the Black Flowerpiercer, *Diglossa humeralis* Fraser, 1840. The new record presented in this contribution highlights the dispersal capabilities of *M. bonariensis*, revealing that this species is able to colonize a city at high altitude.

## Materials and methods

In the campus of the Pontificia Universidad Católica del Ecuador (PUCE), Quito, Ecuador (0°12'40"S, 78°29'28"W), we observed two juveniles of *M. bonariensis* interacting with *Zonotrichia capensis* Müller, 1776, between 11 April and 15 May 2015. One of the juveniles of *M. bonariensis* was larger and more developed than the other, and each was associated with a different *Z. capensis* pair. We observed the juveniles of *M. bonariensis* displaying food begging behaviors to adult *Z. capensis* (which included chasing the sparrows on the ground), and vocalizing intensely on bushes and tree branches. The adults of *Z. capensis* were observed feeding the Shiny Cowbirds on several occasions (Fig. 3a, b) but they were not observed feeding fledglings of their own species.

## Taxon treatments

### Molothrus bonariensis

Gmelin, 1789

#### Materials

- **Type status:** Other material. **Occurrence:** recordedBy: Verónica Crespo-Pérez; C. Miguel Pinto; individualCount: 2; lifeStage: juvenile; **Taxon:** genus: Molothrus; specificEpithet: bonariensis; scientificNameAuthorship: Gmelin, 1789; vernacularName: Shiny Cowbird, Vaquero brilloso; **Location:** continent: South America; country: ECUADOR; stateProvince: Pichincha; municipality: Quito; verbatimElevation: 2800 m; verbatimCoordinateSystem: decimal degrees; decimalLatitude: -0.2111111; decimalLongitude: -78.49111; geodeticDatum: WGS84; **Identification:** identifiedBy: Tjitte de Vries; **Event:** eventDate: 2015-04-11; habitat: urban university campus

#### Diagnosis

The observed individuals of Shiny Cowbird matched the morphological and behavioral characterstics of the species (Fig. 3a, b, c). The most remarkable characteristic was the dull grayish coloration on the dorsum, but paler on the venter and throat, and the greyish white eyebrows (Hilty and Brown 1986, Restall et al. 2006). For Ecuador, three subspecies have been documented: *M. b. aequatorialis* in the north, *M. b. occidentalis* in the south, and *M. b. riparius* in the east (Restall et al. 2006). The juveniles that we observed have a clear coloration (Fig. 3a, b) that matches that of the subspecies *M. b. occidentalis*, and not that of *M. b. aequatorialis—* which is darker*—* as would be expected for the location of Quito in the north of the country. Nevertheless, juvenile coloration might not match that of adults and more studies should be conducted for subspecific assignment perhaps using an integrative taxonomic approach combining molecular, morphological and biogeographic information.

## Identification Keys

### Key to the species of *Molothrus*

**Table:** 

1	Male and female weight more than 100 g	*Molothrus oryzivorus*
–	Male and female weight less than 100 g	2
2	Males iridescent black with a brown head	*Molothrus ater*
–	Males iridescent black without contrasting coloration of head	3
3	Males with red eyes during breeding season	*Molothrus aeneus*
–	Males with brown eyes during breeding season	4
4	Both sexes are dimorphic in coloration, males have a violet gloss	*Molothrus bonarensis*
–	Coloration of both sexes is similar, and males are less glossy than *M. bonarensis*	*Molothrus rufoaxillaris*

## Discussion

In recent years the Shiny Cowbird has colonized the Caribbean and the east coast of the United States (Fig. 1). Particularly in Ecuador, this species seemed to be restricted to below ca. 2,000m asl (Hilty and Brown 1986, Ridgely and Greenfield 2001, Restall et al. 2006, Lowther 2011), with isolated higher altitude records (Fjeldsa and Krabbe 1990, Ridgely and Greenfield 2001, Xeno-canto Foundation 2012) (Fig. 2), and had never before been reported in the high-altitude city of Quito. Surely, this inability to colonize the city was puzzling since it has a very long history of habitat alteration, and one of the Shiny Cowbird's most common hosts, *Z. capensis*, does well in high elevations and is abundant in Quito (Carrion 2001, Ridgely and Greenfield 2001). Even though there is strong evidence suggesting habitat fragmentation is the main driver of *M. bonariensis* range expansions (Arendt and Vargas Mora 1984, Post et al. 1993, Hansen et al. 2005) we believe that in this case, climate change might be playing an important role as well. Recent climate warming may have relaxed climatic conditions allowing this species to expand towards higher elevations. There is, in fact, evidence of temperature increases in the Ecuadorian Andes (e.g., Vuille et al. 2008), which may be related to range expansions such as the one presented in this contribution. Climate induced distributional shifts have been fairly well documented especially in species with high dispersal ability like birds, insects and marine invertebrates (Parmesan 2006). Several authors have reported range expansions of species towards higher altitudes (Paulson 2001, Parmesan 2006, Moritz et al. 2008, Chen et al. 2009, Chen et al. 2011). Along the Andes there are several examples of upslope expansion of distributions of plants and birds (Weng et al. 2007, Feeley et al. 2010, Forero-Medina et al. 2011, Morueta-Holme et al. 2015). Also, according to recent evidence, tropical species respond more strongly than temperate ones to warming temperatures, with range shifts that match local temperature increase more closely than in temperate-zone montane species (Freeman and Freeman 2014).

Avian brood parasites, including cuckoos and cowbirds, often reduce the reproductive success of their hosts (Tuero et al. 2007). Costs associated with such parasitism promote the evolution of host antiparasitic defenses – such as responding aggressively towards cowbirds to prevent them from gaining access to the nest, burying or ejecting parasite eggs, or abandoning parasitized nests – and create a coevolutionary arms race between hosts and parasites (Astie and Reboreda 2005). Nevertheless, a number of hosts, even commonly parasitized hosts like the Rufous-collared sparrow, have not evolved antiparasite defenses, despite the associated fitness costs, due probably to morphological or ecological constraints (Carro and Fernández 2013). In addition, in the case of invasive parasites, native hosts may be naïve to the parasite and lack defenses or appropriate mechanisms to counteract its negative effects (Taraschewski 2006, Fassbinder-Orth et al. 2013). The level of threat posed by invasive Cowbirds on native bird communities apparently depends on the local species present (i.e., whether they lack defenses against brood parasitism), and is closely related to other human disturbances, mainly habitat fragmentation (Rothstein and Peer 2005, Peer et al. 2013. In the specific case of the Shiny Cowbird, several studies have found negative effects of this parasite, especially for already endangered and/or endemic bird species (Post 2011, Price et al. 2011, Domínguez et al. 2014). In fact, there is evidence that suggests that Shiny Cowbird parasitism, coupled with habitat loss, almost drove the Pale-headed Brush-Finch, *Atlapetes pallidiceps* Sharpe, 1900, a critically endangered endemic to south-central Ecuador, to extinction (Oppel et al. 2004, Krabbe et al. 2010). Therefore, the Shiny Cowbird range expansion presented in this study may be of concern, especially for native, endemic, or endangered species that may be parasitized by this species. More studies are urgently needed to assess the threat posed by this invasive species to native avian fauna in the high Andes in order to guide management or control measures for this invasive brood parasite.

## Supplementary Material

XML Treatment for Molothrus bonariensis

## Figures and Tables

**Figure 3a.**
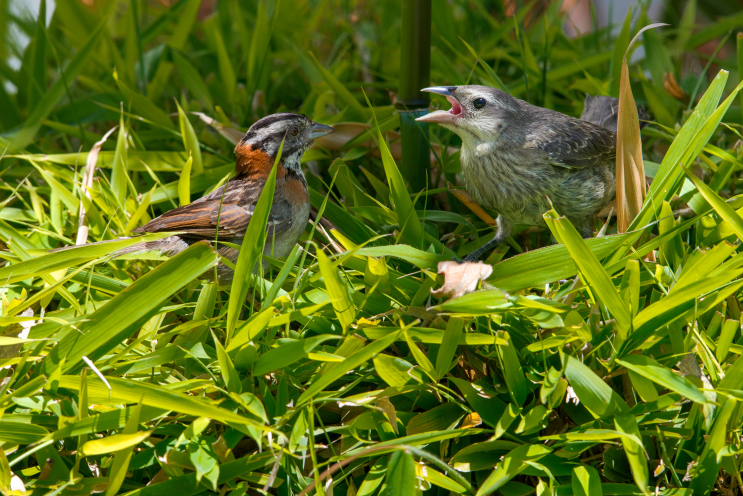
Foster *Zonotrichia capensis* parent (left) approaching juvenile Shiny cowbird (right). Photographed with a Nikon D800 camera and a Nikon AF-S NIKKOR 400mm f/2.8G ED VR lens by RDJ.

**Figure 3b.**
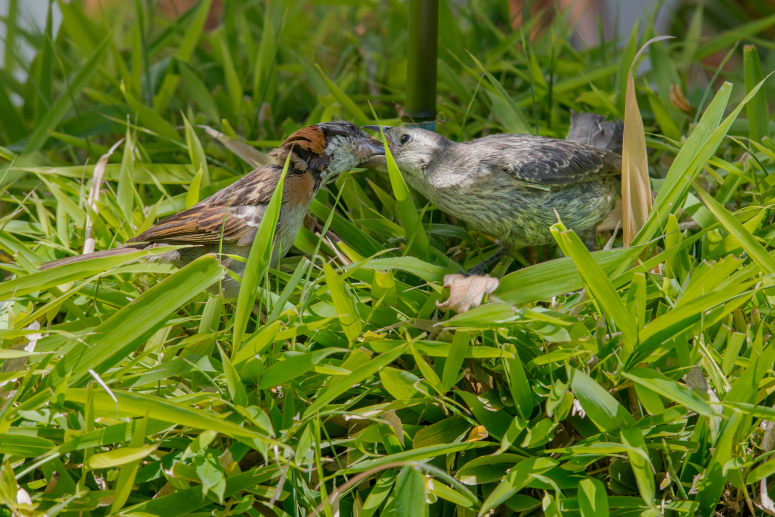
Foster *Zonotrichia capensis* parent (left) feeding juvenile Shiny cowbird (right). Photographed with a Nikon D800 camera and a Nikon AF-S NIKKOR 400mm f/2.8G ED VR lens by RDJ.

**Figure 3c.**
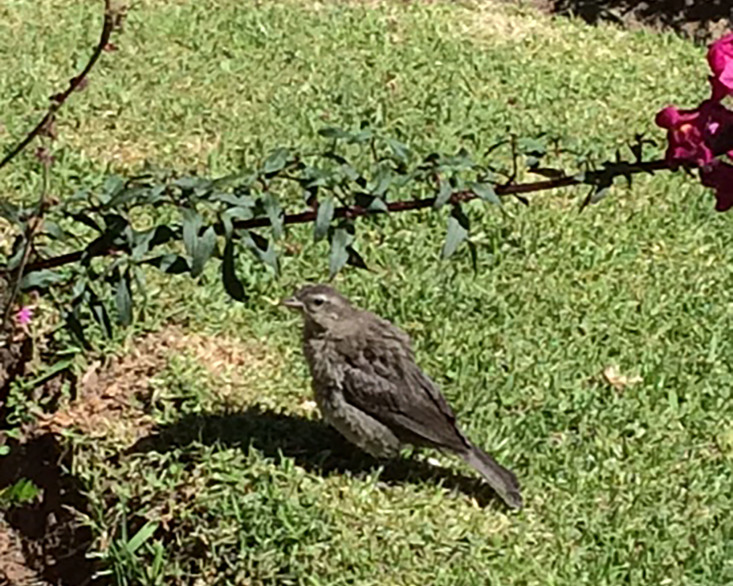
Juvenile of Shiny Cowbird showing the grey coloration, eye band and paler underparts. Photographed with a cell phone by VCP.

## References

[B3127463] Arendt W. J., Vargas Mora T. A. (1984). Range Expansion of the Shiny Cowbird in the Dominican Republic. Journal of Field Ornithology.

[B2209299] Astie Andrea A., Reboreda Juan C. (2005). Creamy-bellied Thrush defenses against Shiny Cowbird brood parasitism. The Condor.

[B3127273] Balderrama J. A. (2006). Diversidad, endemismo y conservación de la ornitofauna del Parque Nacional Tunari (Cochabamba, Bolivia). Ecologia de Bolivia.

[B2209360] NatureServe Bird Life International and Bird Species Distribution Maps of the World. http://maps.iucnredlist.org/map.html?id=22724345.

[B2209369] Carrion J. M. (2001). Aves de Quito, Retratos y encuentros.

[B2209378] Carro Mariana E., Fernández Gustavo J. (2013). Can nest predation explain the lack of defenses against cowbird brood parasitism in the Rufous-collared Sparrow (*Zonotrichia
capensis*)?. The Auk.

[B2209388] Chen I-Ching, Hill Jane K., Shiu Hau-Jie, Holloway Jeremy D., Benedick Suzan, Chey Vun Khen, Barlow Henry S., Thomas Chris D. (2011). Asymmetric boundary shifts of tropical montane Lepidoptera over four decades of climate warming. Global Ecology and Biogeography.

[B2215031] Chen I. - C., Shiu H. - J., Benedick S., Holloway J. D., Chey V. K., Barlow H. S., Hill J. K., Thomas C. D. (2009). Elevation increases in moth assemblages over 42 years on a tropical mountain. Proceedings of the National Academy of Sciences.

[B3127659] Domínguez M., Reboreda J. A., Mahler B. (2014). Impact of Shiny Cowbird and botfly parasitism on the reproductive success of the globally endangered Yellow Cardinal Gubernatrix cristata. Bird Conservation International.

[B2209402] Fassbinder-Orth Carol A., Barak Virginia A., Brown Charles R. (2013). Immune Responses of a Native and an Invasive Bird to Buggy Creek Virus (Togaviridae: Alphavirus) and Its Arthropod Vector, the Swallow Bug (*Oeciacus
vicarius*). PLoS ONE.

[B3127303] Feeley Kenneth J., Silman Miles R., Bush Mark B., Farfan William, Cabrera Karina Garcia, Malhi Yadvinder, Meir Patrick, Revilla Norma Salinas, Quisiyupanqui Mireya Natividad Raurau, Saatchi Sassan (2010). Upslope migration of Andean trees. Journal of Biogeography.

[B3171740] Fjeldsa J., Krabbe N. (1990). Birds of the High Andes: A Manual to the Birds of the Temperate Zone of the Andes and Patagonia, South America.

[B3127293] Forero-Medina German, Terborgh John, Socolar S. Jacob, Pimm Stuart L. (2011). Elevational Ranges of Birds on a Tropical Montane Gradient Lag behind Warming Temperatures. PLoS ONE.

[B2209412] Freeman B. G., Freeman A. M. C. (2014). Rapid upslope shifts in New Guinean birds illustrate strong distributional responses of tropical montane species to global warming. Proceedings of the National Academy of Sciences.

[B3127473] Hansen Andrew J., Knight Richard L., Marzluff John M., Powell Scott, Brown Kathryn, Gude Patricia H., Jones Kingsford (2005). Effects of exurban development on biodiversity: patterns, mechanisms, and research needs. Ecological Applications.

[B2209422] Hilty SH, Brown WL (1986). A guide to the birds of Colombia.

[B3171769] Jaramillo A., Burke P. (1999). New world blackbirds: the icterids.

[B2209431] Kluza D. A. (1998). First record of Shiny Cowbird (*Molothrus
bonariensis*) in Yucatán, Mexico. Wilson Bulletin-Morgantown Then Columbus-Ornithological Bulletin.

[B3127669] Krabbe Niels, Juiña Mery, Sornoza Aldo Fernando (2010). Marked population increase in Pale-headed Brush-finch Atlapetes
pallidiceps in response to cowbird control. Journal of Ornithology.

[B2209451] Lowther P. E Shiny Cowbird (Molothrus
bonariensis). Neotropical Birds Online. http://neotropical.birds.cornell.edu/portal/species/overview?p_p_spp=672716.

[B2209460] Lowther P. E. Lists of victims and hosts of the parasitic cowbirds, Version 26 August 2013. http://fieldmuseum.org/sites/default/files/Molothrus_hosts-26aug2013.pdf.

[B2209469] Mason P. (1986). Brood parasitism in a host generalist, the Shiny Cowbird: I. The quality of different species as hosts. The Auk.

[B3171778] Miller L. E. (1917). Field notes on *Molothrus
bonariensis* and *Molothrus
badius*. Bulletin of the American Museum of Natural History.

[B2209479] Moritz C., Patton J. L., Conroy C. J., Parra J. L., White G. C., Beissinger S. R. (2008). Impact of a century of climate change on small-mammal communities in Yosemite National Park, USA. Science.

[B2209491] Morueta-Holme Naia, Engemann Kristine, Sandoval-Acuña Pablo, Jonas Jeremy D., Segnitz R. Max, Svenning Jens-Christian (2015). Strong upslope shifts in Chimborazo's vegetation over two centuries since Humboldt. Proceedings of the National Academy of Sciences.

[B3127680] Oppel S., Schaefer H. M., Schmidt V., Schroder B. (2004). Cowbird parasitism of Pale-headed Brush-finch Atlapetes
pallidiceps: implications for conservation and management. Bird Conservation International.

[B2209503] Parmesan Camille (2006). Ecological and evolutionary responses to recent climate change. Annual Review of Ecology, Evolution, and Systematics.

[B2209513] Paulson Dennis R. (2001). Recent Odonata records from southern Florida - effects of global warming?. International Journal of Odonatology.

[B3127566] Peer Brian D., Rivers James W., Rothstein Stephen I. (2013). Cowbirds, conservation, and coevolution: potential misconceptions and directions for future research. Chinese Birds.

[B3127595] Post William Yellow-shouldered Blackbird (Agelaius xanthomus). http://neotropical.birds.cornell.edu/portal/species/overview?p_p_spp=666636.

[B2209523] Post William, Cruz A., McNair D. B. (1993). The North American Invasion Pattern of the Shiny Cowbird. Journal of Field Ornithology.

[B3127577] Price Melissa R., Lee Valerie A., Hayes William K. (2011). Population status, habitat dependence, and reproductive ecology of Bahama Orioles: a critically endangered synanthropic species. Journal of Field Ornithology.

[B2214905] Restall R, Rodner C, Lentino M (2006). Birds of northern South America, vol. 2: an identification guide..

[B2214914] Ridgely R., Greenfield P. J. (2001). The Birds of Ecuador: Status, Distribution, and Taxonomy.

[B2214957] Ridgely R., Greenfield P. J. (2006). Aves del Ecuador: Guía de campo.

[B3127555] Rothstein Stephen I., Peer Brian D. (2005). Conservation Solutions for Threatened and Endangered Cowbird (Molothrus spp.) Hosts: Separating Fact from Fiction. Ornithological Monographs.

[B2214966] Sandoval L., Sánchez C., Biamonte E., Zook J. R., Sánchez J. E., Martínez D., Loth D., O’Donahoe J. (2010). Recent records of new and rare bird species in Costa Rica. Bulletin of the British Ornithologists’ Club.

[B3171749] Schulenberg T. S., Stotz D. F., Lane D. F., O'Neill J. P., Parker T. A. (2007). Birds of Peru.

[B2214980] Taraschewski H. (2006). Hosts and parasites as aliens. Journal of Helminthology.

[B2214990] Tuero D. T., Fiorini V. D., Reboreda J. C. (2007). Effects of Shiny Cowbird *Molothrus
bonariensis* parasitism on different components of House Wren Troglodytes aedon reproductive success. Ibis.

[B2215000] Vuille Mathias, Francou Bernard, Wagnon Patrick, Juen Irmgard, Kaser Georg, Mark Bryan G., Bradley Raymond S. (2008). Climate change and tropical Andean glaciers: Past, present and future. Earth-Science Reviews.

[B3127283] Weng C., Hooghiemstra H., Duivenvoorden J. F (2007). Response of pollen diversity to the climate-driven altitudinal shift of vegetation in the Colombian Andes. Philosophical Transactions of the Royal Society B: Biological Sciences.

[B3127046] Foundation Xeno-canto Xeno-canto: Sharing bird sounds from around the world. http://www.xeno-canto.org/species/Molothrus-Molothrusbonariensis.

